# Supplementary material for: High-throughput amplicon sequencing of the full-length 16S rRNA gene with single-nucleotide resolution
Source: Nucleic Acids Res. 2019 Jul 3;47(18):e103. doi: 10.1093/nar/gkz569 (PMC6765137; doi:10.1093/nar/gkz569)
Supplement: gkz569_Supplemental_Files [file gkz569_supplemental_files.zip › Supplementary_Document_1.pdf]

Procedure & Checklist - Full-Length 16S Amplification, SMRTbell® Library Preparation and Sequencing

This document contains instructions for amplifying and sequencing a full-length 16S gene from bacterial DNA isolated from metagenomic samples. Tests with mock community samples produced discrete 16S amplicons with adequate yield for library preparation and SMRT sequencing. Data analysis showed good representation of community members in the samples, with low rates of chimerism.

The workflow employs 2 rounds of PCR, the first with universal primer-tailed 16S primers and the second with PacBio Barcoded Universal Primers.

Materials and Kits Needed

Item	Vendor
**Amplification**	
KAPA HiFi HotStart PCR Kit	KAPA Biosciences
27F Primer tailed with universal sequence*	Any Oligo Vendor
1492R Primer tailed with universal sequence**	Any Oligo Vendor
Barcoded Universal Primers	PacBio
**Library Prep**	
SMRTbell Template Prep Kit	Pacific Biosciences
AMPure® PB beads	
**QC Tools**	
Qubit	Invitrogen
BioAnalyzer	Agilent
DNA 12000 Kit or HS DNA Kit	Agilent

The first round of amplification requires user-supplied **16S Primers (Forward and Reverse) Tailed with Universal Sequences**. In addition to universal sequence tails, a 5' block is added, (e.g., 5'NH2-C6) to ensure that carry-over amplicons from first round PCR are not ligated to the SMRTbell adapters in subsequent steps. HPLC purification is recommended. The structure of the oligos is 5' Block - universal sequence - 16S primer:

Primer	5' Block	Universal Sequence	16S PRIMER
***27F**:	*/5AmMC6/*	gcagtcgaacatgtagctgactcaggtcac	AGRGTTYGATYMTGGCTCAG
****1492R**:	*/5AmMC6/*	tggatcacttgtgcaagcatcacatcgtag	RGYTACCTTGTTACGACTT

Requirements

1. Use only high-quality primers; damaged bases at the ends of the amplicons can't be repaired by DNA Damage Repair enzymes and will result in low library yield.
2. Primers should be stored at high concentration in buffered solution (e.g., 100 µM primer in 10 mM Tris pH8), and should not be subjected to repeated freeze thaws.

Extracting Genomic DNA from Metagenomic Samples

Due to the harsh lysis methods required for some organisms, it may be difficult to extract high quality, intact genomic DNA (gDNA) from metagenomic samples which may slightly impact yield for some types of samples. However, for most samples, quality and yield are likely sufficient for full-length 16S amplification.

It is important to note that the relative abundance of gDNA for community members may be impacted by the extraction method used. An improved method for isolating DNA of the human intestinal microbiome is described here. Samples generated with this method tend to provide good sequencing data and coverage.

Multiplexing of 16S Amplicons

For studies targeting a single consensus sequence per sample, or for identification of relatively abundant organisms in non-complex metagenomic communities, 16S amplicons may be multiplexed to utilize the complete capacity of a SMRT Cell.

PacBio has designed a set of 16-bp barcodes for optimal discrimination with SMRT Sequencing. Barcodes may be incorporated into SMRTbell libraries through amplification. There are 2 options using barcoded PCR primers:

1. **Forward and reverse universal-tail 16S primers (27F and 1492R), with the PacBio Barcoded Universal Primers** (96-well plate kit): This method is highly recommended. A universal tail is added to the forward and reverse 16S primers, and barcodes are added during second round of amplification, with PacBio Barcoded Universal forward and reverse primers. For more background on PacBio Universal Primers, see *Preparing SMRTbell™ Libraries using PacBio® Barcoded Universal Primers for Multiplex SMRT® Sequencing.* This procedure describes how to use Barcoded Universal Primers.
2. **Barcoded 16S (27F and 1492R) Primers**: Both forward and reverse 16S primers are tailed with PacBio barcode sequences. Guidelines and spreadsheets for primer ordering may be found in the SMRT® Analysis Barcoding Overview (v5.1.0). Sequences for 384 PacBio barcodes are provided. If using this option, use the same condition outlined in the "first-round" PCR and eliminating the "second-round" PCR.

16S Amplification

In tests with mock community samples, this protocol yields 500 ng to 1 µg of amplicon product from 5 ng to as little as 50 pg of template DNA. Yield may be lower for some samples with a large amount of contaminating non-bacterial gDNA.

Multiplexing with PacBio Barcoded Universal Primers

STEP	✓	First Round Amplification	Notes
		In this section, you will need the following:<br>● 16S Primers tailed with universal sequences (see page 1 for structure and ordering recommendations)<br>● KAPA HiFi Hot Start PCR Kit	
1		Prepare a PCR master mix with the appropriate volumes of all common components (except water, if varying volumes of template DNA are used). Insure that reagents are thawed and mixed prior to preparing the master mix.	

Component	Volume (25 µL Reaction)
PCR-grade Water	As Required for 25 µL reaction
5X KAPA HiFi Buffer	5 µL
10 mM dNTPs	0.75 µL
10 µM 27F Forward Primer	0.75 µL
10 µM 1492R Reverse Primer	0.75 µL
Template DNA	25 pg – 2.5 ng*
1U/ µL KAPA HiFi Hot Start DNA Polymerase	0.75 µL
Total volume	25 µL

*If PCR yield is below 500 ng, or if the sample is suspected of having a large amount of contaminating large genome DNA, input may be increased to 5 ng. Lower inputs may be used for multiplexed samples. Higher inputs of clean metagenomic DNA may lead to increased chimera formation.

For 2.5 ng input DNA, cycle using the following conditions:

Step	Temp	Time
1	95 °C	30 seconds
2	57 °C	30 seconds
3	72 °C	60 seconds
4	Repeat steps 1 to 3 for a total of 20 cycles*	

*A higher number of cycles may be required for contaminated samples or very low input samples. For input of 0.25 ng, use up to 23 cycles. For 25 pg DNA, use up to 27 cycles.

STEP	✓	First Round Amplification	Notes
2		QC PCR product from first-round PCR prior to taking samples into the second-PCR. For best results, normalize samples going into the second-round PCR step:<br>● Load samples on an agarose gel for visual inspection or quantitate using the Agilent® 2200 or 4200 TapeStation instrument or an Agilent 2100 Bioanalyzer System.<br>● The amount of PCR product for use in the second round of PCR should be roughly equimolar across samples. If some samples appear weak or undetectable, use more volume in the second-round, or re-amplify with a higher number of cycles.	

STEP	✓	Second Round Amplification	Notes
		In this section, you will need the following:<br>● PacBio Barcoded Universal F/R Primers Plate – 96<br>● KAPA HiFi Hot Start PCR Kit	
1		Prepare a PCR master mix with the appropriate volumes of all common components (except water, if varying volumes of template DNA are used). Insure that reagents are thawed and mixed prior to preparing the master mix. (see tables below)	
2		Proceed to the next section for AMPure PB bead purification of amplicons. Note that recovery of clean samples from AMPure PB bead purification should be between 80-100%. However, yields will be lower for samples with smaller contaminating fragments or primer dimers.<br><br>It is important to check the size and purity of amplicon samples before preparing SMRTbell libraries for sequencing. If aberrant products are present, they must be removed prior to, or during, library preparation. If present in the final library, shorter contaminants may represent a substantial percentage of the sequencing reads.	

Step 1 tables:

Component	Volume (25 µL Reaction)
PCR-grade Water	As Required for 25 µL reaction
5X KAPA HiFi Buffer	5 µL
10 mM dNTPs	0.75 µL
2 µM Barcoded Universal Primers	3.75 µL
First Round PCR Product	1 - 2 ng*
1U/ µL KAPA HiFi Hot Start DNA Polymerase	0.5 µL
Total volume	25 µL

*Higher input may lead to increased chimera formation. Use higher volumes for samples with weak or undetectable bands on agarose gel QC.

Cycle using the following conditions:

Step	Temp	Time
1	95 °C	30 seconds
2	57 °C	30 seconds
3	72 °C	60 seconds
4	Repeat steps 1 to 3 for a total of 20 cycles*	

*A higher number of cycles may be required for contaminated samples or very low input samples.

STEP		AMPure PB Bead Purification	Notes
1		Determine the sample volume. Add **0.60X v**olume **(or 0.50X** volume for removal of non-specific products <1 kb.**)** of AMPure PB beads to the sample. For 25 µL PCR reactions, add an additional 25 µL of high quality water or EB, then add the appropriate volume AMPure beads to allow for minor pipetting errors with AMPure beads.	
2		Mix the bead/DNA solution thoroughly.	
3		Quickly spin down the tube (for 1 second) to collect the beads	
4		Allow the DNA to bind to beads by mixing in a VWR® vortex mixer at 2000 rpm for 10 minutes at room temperature. Note that the bead/DNA mixing is critical to yield.	
5		Spin down the tube (for 1 second) to collect the beads.	
6		Place the tube in a magnetic bead rack until the beads collect to the side of the tube and the solution appears clear. The actual time required to collect the beads to the side depends on the volume of beads added.	
7		With the tube still on the magnetic bead rack, slowly pipette off cleared supernatant and save in another tube. Avoid disturbing the bead pellet.<br>If the DNA is not recovered at the end of this Procedure, you can add equal volumes of AMPure PB beads to the saved supernatant and repeat the AMPure PB bead purification steps to recover the DNA.	
8		Wash beads with freshly prepared 70% ethanol.<br>Note that 70% ethanol is hygroscopic and should be prepared FRESH to achieve optimal results. Store 70% ethanol in a tightly capped polypropylene tube for no more than 3 days.<br>– Do not remove the tube from the magnetic rack.<br>– Use a sufficient volume of 70% ethanol to fill the tube (1.5 mL for 1.5 mL tube or 2 mL for 2 mL tube). Slowly dispense the 70% ethanol against the side of the tube opposite the beads. Let the tube sit for 30 seconds.<br>– Do not disturb the bead pellet.<br>– After 30 seconds, pipette and discard the 70% ethanol.	
9		Repeat step 8 above.	
10		Remove residual 70% ethanol and dry the bead pellet.<br>– Remove tube from magnetic bead rack and spin to pellet beads. Both the beads and any residual 70% ethanol will be at the bottom of the tube.<br>– Place the tube back on magnetic bead rack.<br>– Pipette off any remaining 70% ethanol.	
11		Check for any remaining droplets in the tube. If droplets are present, repeat step 10.	
12		Remove the tube from the magnetic bead rack and allow beads to air-dry (with the tube caps open) for 60 seconds.	
13		Add **37- 38 µL** of Elution Buffer to the beads to elute the DNA<br>– Mix until homogeneous.<br>– Vortex for 1-2 minutes at 2000 rpm.<br>– Spin the tube down to pellet beads, then place the tube back on the magnetic bead rack.<br>– Carefully collect the eluted sample.<br>– Discard the beads.	
14		Verify the DNA amount and concentration using a Qubit quantitation platform. – Measure the DNA concentration using a Qubit fluorometer.<br>– Using 1 µL of the eluted sample, make a 1:10 dilution in EB.<br>– Use 1 µL of this 1:10 dilution to measure the DNA concentration using a Qubit dsDNA BR Assay kit and the dsDNA HS Assay kit according to the manufacturer's recommendations.	
15		Perform qualitative and quantitative analysis using a Bioanalyzer instrument with the DNA 12000 Kit.	

STEP	✓	Pooling Barcoded Amplicons	Notes
		In this section, barcoded amplicons are pooled for library construction.	
1		Using sample information from the Qubit and BioAnalyzer, determine the molarity of the PCR products, which can be calculated by the following equation: $\frac{\text{concentration in ng/uL}}{(660\ \text{g/mol} \times 1600\ \text{bp})} \times 10^6 = \text{concentration in nM}$	
2		Pool equal molar quantities of the amplicons in a clean LoBind microcentrifuge tube. The total combined mass must be > 500 ng.	
3		Proceed to SMRTbell Template Preparation below. You need at least 500 ng for library construction.	

Repair DNA Damage

Use the following table to repair any DNA damage.

1. In a LoBind microcentrifuge tube, add the following reagents. For more than 1 sample, prepare a pre-mix of DNA Damage Repair Buffer, NAD+, ATP high, and dNTP, and add 11 µL of pre-mix per sample.

Reagent	Tube Cap Color	Stock Conc.	Volume	Final Conc.	✓	Notes
Pooled PCR Product			37 µL			
DNA Damage Repair Buffer		10X	5.0 µL	1X		
NAD+		100X	0.5 µL	1X		
ATP high		10 mM	5.0 µL	1 mM		
dNTP		10 mM	0.5 µL	0.1 mM		
DNA Damage Repair Mix			2.0 µL			
Total Volume			50.0 µL			

2. Mix the reaction well by pipetting or flicking the tube.
3. Spin down contents of tube with a quick spin in a microfuge.
4. Incubate at 37ºC for 60 minutes or longer, up to 150 minutes, then return the reaction to 4°C for 1 minute.

Repair Ends

Use the following table to prepare your reaction then purify the DNA.

Reagent	Tube Cap Color	Stock Conc.	Volume	Final Conc.	✓	Notes
DNA (Damage Repaired)			50 µL	-		
End Repair Mix	Red	20 X	2.0 µL	1X		
Total Volume			52.0 µL	-		

1. Mix the reaction well by pipetting or flicking the tube.
2. Spin down contents of tube with a quick spin in a microfuge.
3. Incubate at 25ºC for 5 minutes, return the reaction to 4ºC. Do not incubate the End Repair reaction longer than 5 minutes. Proceed directly to the next step.

STEP	✓	Purify DNA	Notes
**1**		Add **0.6X** volume or **31.5 µL** of AMPure PB beads of AMPure PB beads to the End-Repair reaction.	
**2**		Mix the bead/DNA solution thoroughly.	
**3**		Quickly spin down the tube (for 1 second) to collect the beads. Do not pellet beads.	
**4**		Allow the DNA to bind to beads by shaking in a VWR vortex mixer at 2000 rpm for 10 minutes at room temperature.	
**5**		Spin down the tube (for 1 second) to collect beads.	
**6**		Place the tube in a magnetic bead rack to collect the beads to the side of the tube.	
**7**		Slowly pipette off cleared supernatant and save (in another tube). Avoid disturbing the bead pellet.	
**8**		Wash beads with freshly prepared 70% ethanol.	
**9**		Repeat step 8 above.	
**10**		Remove residual 70% ethanol and dry the bead pellet.<br>– Remove tube from magnetic bead rack and spin to pellet beads. Both the beads and any residual 70% ethanol will be at the bottom of the tube.<br>– Place the tube back on magnetic bead rack.<br>– Pipette off any remaining 70% ethanol.	
**11**		Check for any remaining droplets in the tube. If droplets are present, repeat step 10.	
**12**		Remove the tube from the magnetic bead rack and allow beads to air-dry (with tube caps open) for 30 to 60 seconds.	
**13**		Elute the DNA off the beads:<br>– Add **31-32 µL** Elution Buffer, mix until homogenous<br>– Vortex for 1-2 minute at 2000 rpm<br>– Spin the tube down to pellet beads, then place the tube back on the magnetic bead rack.<br>– Carefully collect the eluted sample.<br>– Discard the beads.	
**14**		Optional: Verify your DNA amount and concentration using a Nanodrop or Qubit quantitation platform, as appropriate.<br>Actual recovery per µL and total available sample material: ______<br>Note that typical yield at this point of the process (following End- Repair and one AMPure PB bead purification) is approximately 80-100% of the total starting material going into the Damage Repair reaction.	
**15**		The End-Repaired DNA can be stored overnight at 4ºC, or at -20ºC for longer duration.	

Prepare Blunt-Ligation Reaction

Use the following table to prepare your blunt-ligation reaction:

1. In a LoBind microcentrifuge tube (on ice), add the following reagents in the order shown.
   - Template Prep Buffer and ATP low may be pre-mixed; blunt adapter and ligase should be added separately in the order listed below and mixed well prior to the next step.
   - The adapter concentration below is appropriate for the input amounts from the table on page 2. If a higher amount of input DNA is being used, adjust the adapter concentration accordingly to minimize double-insert SMRTbell templates; a 30-50X molar excess of adapter is recommended.

Reagent	Tube Cap Color	Stock Conc.	Volume	Final Conc.	✓	Notes
DNA (End Repaired)			32 µl			
Annealed Blunt Adapter (20 µM)		20 µM	1.0 µL	0.5 µM		
Mix before proceeding						
Template Prep Buffer		10 X	4.0 µL	1X		
ATP low		1 mM	2.0 µL	0.05 mM		
Mix before proceeding						
Ligase		30 U/µL	1.0 µL	0.75 U/µL		
Total Volume			40.0 µL			

2. Mix the reaction well by pipetting or flicking the tube.
3. Spin down contents of tube with a quick spin in a microfuge.
4. Incubate at 25°C for 15 minutes to 24 hours (overnight).
5. Incubate at 65°C for 10 minutes to inactivate the ligase, then return the reaction to 4°C.

Add Exonucleases to Remove Failed Ligation Products

Reagent	Tube Cap Color	Stock Conc.	✓	Volume
Ligated DNA				40 µL
Mix reaction well by pipetting				
ExoIII		100.0 U/µL		0.5 µL
ExoVII		10.0 U/µL		0.5 µL
Total Volume				41.0 µL

1. Mix the reaction well by pipetting or flicking the tube.
2. Spin down contents of tube with a quick spin in a microfuge.
3. Incubate at 37°C for 1 hour, then return the reaction to 4°C. You must proceed with purification after this step.

Purify SMRTbell® Templates

There are 3 purification steps using the appropriate volume of AMPure PB beads for each step, see table below. For more stringent purification from shorter contaminants, 0.5X or 0.45X AMPure beads may be used in the final steps.

STEP	✓	Purify SMRTbell Templates - First Purification	Notes
**1**		Add **0.6X** volume or **24.6 µL** AMPure PB beads to the exonuclease-treated reaction, following the chart below.	
**2**		Mix the bead/DNA solution thoroughly.	
**3**		Quickly spin down the tube (for 1 second) to collect the beads. Do not pellet beads.	
**4**		Allow the DNA to bind to beads by shaking in a VWR vortex mixer at 2000 rpm for 10 minutes at room temperature.	
**5**		Spin down the tube (for 1 second) to collect beads.	
**6**		Place the tube in a magnetic bead rack to collect the beads to the side of the tube.	
**7**		Slowly pipette off cleared supernatant and save (in another tube). Avoid disturbing the bead pellet.	
**8**		Wash beads with freshly prepared 70% ethanol.	
**9**		Repeat step 8 above.	
**10**		Remove residual 70% ethanol and dry the bead pellet.<br>– Remove tube from magnetic bead rack and spin to pellet beads. Both the beads and any residual 70% ethanol will be at the bottom of the tube.<br>– Place the tube back on magnetic bead rack.<br>– Pipette off any remaining 70% ethanol.	
**11**		Check for any remaining droplets in the tube. If droplets are present, repeat step 10.	
**12**		Remove the tube from the magnetic bead rack and allow beads to air-dry (with tube caps open) for 30 to 60 seconds.	
**13**		Elute the DNA off the beads:<br>– Add 50 µL Elution Buffer, mix until homogenous<br>– Vortex for 1-2 minute at 2000 rpm<br>– Spin the tube down to pellet beads, then place the tube back on the magnetic bead rack.<br>– Carefully collect the eluted sample.<br>– Discard the beads.<br>[illegible]	
**14**		The eluted DNA in **50 µL** Elution Buffer should be taken into the second AMPure PB bead purification step.	

STEP	✓	Purify SMRTbell Templates - Second Purification	Notes
1		Add **0.6X** volume or **30 µL** AMPure PB beads to the exonuclease-treated reaction, following the chart below.	
2		Mix the bead/DNA solution thoroughly.	
3		Quickly spin down the tube (for 1 second) to collect the beads. Do not pellet beads.	
4		Allow the DNA to bind to beads by shaking in a VWR vortex mixer at 2000 rpm for 10 minutes at room temperature.	
5		Spin down the tube (for 1 second) to collect beads.	
6		Place the tube in a magnetic bead rack to collect the beads to the side of the tube.	
7		Slowly pipette off cleared supernatant and save (in another tube). Avoid disturbing the bead pellet.	
8		Wash beads with freshly prepared 70% ethanol.	
9		Repeat step 8 above.	
10		Remove residual 70% ethanol and dry the bead pellet.<br>– Remove tube from magnetic bead rack and spin to pellet beads. Both the beads and any residual 70% ethanol will be at the bottom of the tube.<br>– Place the tube back on magnetic bead rack.<br>– Pipette off any remaining 70% ethanol.	
11		Check for any remaining droplets in the tube. If droplets are present, repeat step 10.	
12		Remove the tube from the magnetic bead rack and allow beads to air-dry (with tube caps open) for 30 to 60 seconds.	
13		Elute the DNA off the beads:<br>– Add 50 µL Elution Buffer, mix until homogenous<br>– Vortex for 1-2 minute at 2000 rpm<br>– Spin the tube down to pellet beads, then place the tube back on the magnetic bead rack.<br>– Carefully collect the eluted sample.<br>– Discard the beads.	
14		The eluted DNA in **50 µL** Elution Buffer should be taken into the second AMPure PB bead purification step.	

STEP	✓	Purify SMRTbell Templates - Final Purification	Notes
**1**		Add **30 µL** (**0.6X** volume) AMPure PB beads to **50 µL** of eluted DNA from the previous AMPure PB bead purification step.	
**2**		Mix the bead/DNA solution thoroughly.	
**3**		Quickly spin down the tube (for 1 second) to collect the beads. Do not pellet beads.	
**4**		Allow the DNA to bind to beads by shaking in a VWR vortex mixer at 2000 rpm for 10 minutes at room temperature.	
**5**		Spin down the tube (for 1 second) to collect beads.	
**6**		Place the tube in a magnetic bead rack to collect the beads to the side of the tube.	
**7**		Slowly pipette off cleared supernatant and save (in another tube). Avoid disturbing the bead pellet.	
**8**		Wash beads with freshly prepared 70% ethanol.	
**9**		Repeat step 8 above.	
**10**		Remove residual 70% ethanol and dry the bead pellet.<br>– Remove tube from magnetic bead rack and spin to pellet beads. Both the beads and any residual 70% ethanol will be at the bottom of the tube.<br>– Place the tube back on magnetic bead rack.<br>– Pipette off any remaining 70% ethanol.	
**11**		Check for any remaining droplets in the tube. If droplets are present, repeat step 10.	
**12**		Remove the tube from the magnetic bead rack and allow beads to air-dry (with tube caps open) for 30 to 60 seconds.	
**13**		Elute the DNA off the beads:<br>– Add **10 µL** Elution Buffer, mix until homogenous<br>– Vortex for 1-2 minute at 2000 rpm<br>– Spin the tube down to pellet beads<br>– Place the tube back on the magnetic bead rack and carefully collect the eluted sample.<br>– Discard the beads.	
**14**		Verify the DNA amount and concentration with either a Nanodrop or Qubit quantitation reading.	
**15**		Perform qualitative analysis using a Bioanalyzer instrument. Note that typical DNA yield, at this point of the process, can range from **15-50%** of the total starting material going into the DNA Damage Repair reaction (depending on the purity and quality of input DNA).	

DNA Control Complex Dilution

The PacBio DNA Control Complex is required for this step. Dilute the DNA Control Complex according to the volumes and instructions specified in SMRT Link Sample Setup.

Anneal and Bind SMRTbell® Templates

For primer annealing, follow the instructions in SMRT Link Sample Setup.

For polymerase binding, follow the instructions in SMRT Link Sample Setup.

Sequencing

We recommend performing loading titrations to determine the appropriate loading concentration. For more information, refer to *Quick Reference Card - Diffusion Loading and Pre-Extension Time Recommendations for the Sequel System*.

Revision History (Description)	Version	Date
Initial release.	01	June 2018

For Research Use Only. Not for use in diagnostic procedures. © Copyright 2016 - 2018, Pacific Biosciences of California, Inc. All rights reserved. Information in this document is subject to change without notice. Pacific Biosciences assumes no responsibility for any errors or omissions in this document. Certain notices, terms, conditions and/or use restrictions may pertain to your use of Pacific Biosciences products and/or third party products. Please refer to the applicable Pacific Biosciences Terms and Conditions of Sale and to the applicable license terms at http://www.pacificbiosciences.com/licenses.html. Pacific Biosciences, the Pacific Biosciences logo, PacBio, SMRT, SMRTbell, Iso-Seq ,and Sequel are trademarks of Pacific Biosciences. BluePippin and SageELF are trademarks of Sage Science, Inc. NGS-go and NGSengine are trademarks of GenDx. FEMTO Pulse and Fragment Analyzer are trademarks of Advanced Analytical Technologies. All other trademarks are the sole property of their respective owners.
