# Supplementary material for: High-throughput amplicon sequencing of the full-length 16S rRNA gene with single-nucleotide resolution
Source: Nucleic Acids Res. 2019 Jul 3;47(18):e103. doi: 10.1093/nar/gkz569 (PMC6765137; doi:10.1093/nar/gkz569)
Supplement: gkz569_Supplemental_Files [file gkz569_supplemental_files.zip › Supplementary_Document_2.pdf]

Procedure & Checklist - Preparing Amplicon Libraries using PacBio® Barcoded Adapters for Multiplex SMRT® Sequencing

Before You Begin

This document describes methods for generating Amplicon libraries using PacBio barcoded adapters.

The procedure is a modification to the standard library preparation procedure in that samples must first go through a single tube End-Repair and Ligation reaction. After ligation, samples (depending on the desired multiplex level) are pooled in equimolar quantities for DNA damage repair, followed by treatment with Exo III and VII.

The workflow below summarizes this procedure.

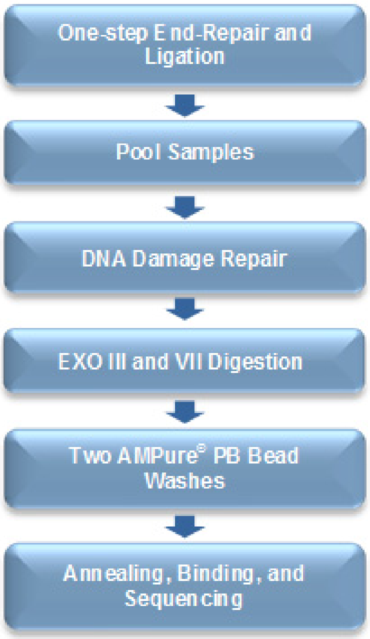

To perform this procedure, you must have the PacBio:

- SMRTbell Barcoded Adapter Complete Prep Kit - 96
  - Barcoded Adapter Plate - 96
  - SMRTbell Barcoded Adapter Prep Kit
- SMRTbell DNA Damage Repair Kit
- DNA/Polymerase Binding Kit
- AMPure® PB Kit
- DNA Sequencing Kit
- SMRT Cells for standard sequencing

Sample Requirements

The table below summarizes the total input DNA required per multiplex pool

Library Size	Total Input DNA Requirement per Pool
< 1 kb	250 ng
1 - 3 kb	500 ng
3 - 10 kb	1 µg
>10 kb	Barcoding Not Recommended

The input DNA required for each sample depends on the multiplex level. To determine the input amount for End-Repair and Ligation for each sample, divide the total input DNA (using the above table) by the number of samples to be pooled (after the End-repair and Ligation reaction).

For example, for a pool 48 samples for a 3.5 kb library, 21 ng of each sample is required for End-Repair and Ligation (1000 ng / 48). Note: For efficient multiplexing, samples for one pool should be similar in length (+/- 10%) and concentration.

One-Step End-Repair and Ligation Reaction

Each individual sample must first go through this step before pooling. This step repairs the ends of the DNA, followed by ligation with the barcoded adapters in a single-tube reaction.

1. Make a pre-mix of the following reaction and store in ice. Account for any pipetting errors when making a pre-mix

Reagent	Tube Cap Color	Stock Conc.	For 1X	For '___' Samples + Overage	✓	Notes
Template Prep Buffer		10 X	1.0 µL	µL		
ATP high		10 mM	1.0 µL	µL		
dNTP		10 mM	0.1 µL	µL		
End Repair Mix		20X	0.5 µL	µL		
Ligase		30 U/µL	0.3 µL	µL		
H_2O (may be adjusted for sample concentrations)	-		0.6 µL	µL		
Total Volume			3.5 µL	µL		

2. Mix by tapping the tube. Do a quick spin down of the tube. Note that the pre-mix must be used immediately.
3. Transfer 3.5 µL aliquots of the above pre-mix to a 96-well plate or LoBind Microcentrifuge tubes.
4. Prepare the samples by diluting each sample to a total 4 µL so that each sample contains equimolar amounts with each other.

5. Add the template and barcoded adapter in the order listed (mix after each addition) for a total of 10 µL per reaction. Keep reagents on ice at all times. Spin down to ensure the reactions are in the bottom of the wells or tubes.
   If working with less than 96 barcodes, the foil seal of the barcoded adapter plate can be easily pierced with a pipet tip. Any untouched adapter barcodes can be stored at -20°C for future use. Each barcode in the well is for one time use only. See the *User Bulletin - Barcode Plate Mapping* for additional information.

Component	Volume	Final Concentration
Sample	4.0 µL	
Barcoded adapter (20 µM)	2.5 µL	5.0 µM
**Total**	10 µL	

6. Incubate as follows:

Temperature	Time
37ºC	20 minutes
25ºC	15 minutes
65ºC	10 minutes
4ºC	hold

7. After the End-Repair and Ligation reaction, pool the 10 µL reactions to be multiplexed and measure the total volume.
8. Perform AMPure PB bead purification. If necessary, split the total volume in 2 to 3 aliquots for convenience. Use the table below to determine the appropriate concentration of AMPure PB beads to use:

Library Size	X AMPure PB Beads
250 bp	1.8X
500 bp	1.0X
1 -3 kb	0.6X
3 - 10 kb	0.45X

9. Measure the concentration using a Qubit system.

STEP	✓	Purify DNA	Notes
**1**		Add the **appropriate** volume of AMPure PB beads to the pooled End-Repair and Ligation reaction.	
**2**		Mix the bead/DNA solution thoroughly by tapping the tube.	
**3**		Quickly spin down the tube to collect the beads.	
**4**		Allow the DNA to bind to beads by shaking in a VWR vortex mixer at 2000 rpm for 10 minutes at room temperature.	
**5**		Spin down the tube to collect beads.	
**6**		Place the tube in a magnetic bead rack to collect the beads to the side of the tube.	
**7**		Slowly pipette off cleared supernatant and save in another tube. Avoid disturbing the bead pellet.	
**8**		Wash beads with freshly prepared 70% ethanol.	
**9**		Repeat step 8 above.	
**10**		Remove residual 70% ethanol.<br>– Remove tube from magnetic bead rack and spin to pellet beads. Both the beads and any residual 70% ethanol will be at the bottom of the tube.<br>– Place the tube back on the magnetic bead rack.<br>– Pipette off any remaining 70% ethanol.	
**11**		Check for any remaining droplets in the tube. If droplets are present, repeat step 10.	
**12**		Remove the tube from the magnetic bead rack and allow beads to air-dry (with tube caps open) for 30 to 60 seconds.	
**13**		Elute the DNA off the beads in **37 µL** Elution Buffer. Mix until homogeneous, then vortex for 1 minute at 2000 rpm.	
**14**		The eluted DNA can be stored overnight at 4ºC or at -20ºC for longer duration.	

Repair DNA Damage

DNA Damage Repair is recommended for all libraries. Use the following table to prepare the reaction, followed by exonuclease digestion.

Library Size	Total Input DNA Requirement per Pool
< 1 kb	250 ng
1 - 3 kb	500 ng
3 - 10 kb	1 - 5 µg

1. In a LoBind microcentrifuge tube, add the following reagents per pooled library.

Reagent	Tube Cap Color	Stock Conc.	Volume	Final Conc.	✓	Notes
DNA	-		37 µL	-		
DNA Damage Repair Buffer	Red	10 X	5.0 µL	1 X		
NAD+	Red	100 X	0.5 µL	1 X		
ATP high	Red	10 mM	5.0 µL	1 mM		
dNTP	Red	10 mM	0.5 µL	0.1 mM		
DNA Damage Repair Mix	Red	15X	2.0 µL			
H_2O	-		__ µL to adjust to 50.0* µL	-		
Total Volume			50.0 µL	-		

2. Mix the reaction well by pipetting or flicking the tube.
3. Spin down contents of tube with a quick spin in a microfuge.
4. Incubate at 37ºC for 20 minutes, then return the reaction to 4ºC.

Exonuclease Digestion

Add exonuclease to remove failed ligation products.

Reagent	Tube Cap Color	Stock Conc.	✓	Volume
Ligated - Damage Repaired DNA				50 µL
Mix reaction well by pipetting				
ExoIII	●	100.0 U/µL		1.0 µL
ExoVII	●	10.0 U/µL		1.0 µL
Total Volume				52 µL

1. Mix the reaction well by pipetting or flicking the tube.
2. Spin down contents of tube with a quick spin in a microfuge.
3. Incubate at 37ºC for 1 hour, then return the reaction to 4ºC. Do not exceed the 1 hour incubation time. You must proceed with purification after this step.

Purify SMRTbell™ Templates

There are 2 purification steps. The AMPure PB bead concentration for both steps depends on the library size. Use the recommended concentrations shown below:

Library Size	X AMPure PB Beads
250 bp	1.8X
500 bp	1.0X
1 -3 kb	0.6X
3 - 10 kb	0.45X

STEP	✓	Purify SMRTbell™ Templates - First Purification	Notes
1		Add the **appropriate** volume of AMPure PB beads to the exonuclease-treated reaction.	
2		Mix the bead/DNA solution thoroughly.	
3		Quickly spin down the tube (for 1 second) to collect the beads. Do not pellet beads.	
4		Allow the DNA to bind to beads by shaking in a VWR vortex mixer at 2000 rpm for 10 minutes at room temperature.	
5		Spin down the tube (for 1 second) to collect beads.	
6		Place the tube in a magnetic bead rack to collect the beads to the side of the tube.	
7		Slowly pipette off cleared supernatant and save (in another tube). Avoid disturbing the bead pellet.	
8		Wash beads with freshly prepared 70% ethanol.	
9		Repeat step 8 above.	
10		Remove residual 70% ethanol and dry the bead pellet.<br>– Remove tube from magnetic bead rack and spin to pellet beads. Both the beads and any residual 70% ethanol will be at the bottom of the tube.<br>– Place the tube back on magnetic bead rack.<br>– Pipette off any remaining 70% ethanol.	
11		Check for any remaining droplets in the tube. If droplets are present, repeat step 10.	
12		Remove the tube from the magnetic bead rack and allow beads to air-dry (with tube caps open) for 30 to 60 seconds.	
13		Elute the DNA off the beads in **50 µL** of Elution Buffer. Vortex for 1 minute at 2000 rpm.	

STEP	✓	Purify SMRTbell™ Templates - Second Purification	Notes
**1**		Add the **appropriate** volume of AMPure PB beads to the **50 µL** of eluted DNA.	
**2**		Mix the bead/DNA solution thoroughly.	
**3**		Quickly spin down the tube (for 1 second) to collect the beads. Do not pellet beads.	
**4**		Allow the DNA to bind to beads by shaking in a VWR vortex mixer at 2000 rpm for 10 minutes at room temperature.	
**5**		Spin down the tube (for 1 second) to collect beads.	
**6**		Place the tube in a magnetic bead rack to collect the beads to the side of the tube.	
**7**		Slowly pipette off cleared supernatant and save (in another tube). Avoid disturbing the bead pellet.	
**8**		Wash beads with freshly prepared 70% ethanol.	
**9**		Repeat step 8 above.	
**10**		Remove residual 70% ethanol and dry the bead pellet.<br>– Remove tube from magnetic bead rack and spin to pellet beads. Both the beads and any residual 70% ethanol will be at the bottom of the tube.<br>– Place the tube back on magnetic bead rack.<br>– Pipette off any remaining 70% ethanol.	
**11**		Check for any remaining droplets in the tube. If droplets are present, repeat step 10.	
**12**		Remove the tube from the magnetic bead rack and allow beads to air-dry (with tube caps open) for 30 to 60 seconds.	
**13**		Elute the DNA off the beads in **8 - 10 µL** of Elution Buffer.<br>– Mix until homogeneous.<br>– Vortex for 1 minute at 2000 rpm.<br>– Spin the tube down to pellet beads, then place the tube back on the magnetic bead rack.<br>– Carefully collect the eluted sample.<br>– Discard the beads.	
**14**		Verify your DNA amount and concentration on a Bioanalyzer instrument using a High Sensitivity DNA Analysis Kit, or check quantitation using Qubit dsDNA HS Assay Kit. If there is too little sample, estimate the concentration based on 10% yield of input amount into the damage repair step.	

Control Complex Dilution

You must have the PacBio Control Complex for this step. Dilute the Control Complex according to the volumes and instructions specified in the Calculator.

Anneal and Bind SMRTbell™ Templates

Before adding the primer to the SMRTbell template, the primer must go through a melting step at 80ºC. This avoids exposing the sample to heat. The template and primer mix can then be incubated at 20ºC for 30 minutes. Note that you must have the PacBio DNA/Polymerase Kit and use LoBind microcentrifuge tubes for this step.

For polymerase binding, incubation at 30ºC for 30 minutes is sufficient. Instructions for polymerase binding are provided by the calculator.

For more information about using the Binding Calculator, see the Pacific Biosciences *Template Preparation and Sequencing Guide* and *QRC - Annealing and Binding Recommendations*.

Sequence

To prepare for sequencing on the instrument, refer to the *RS Remote Online Help* system or *Pacific Biosciences Software Getting Started Guide* for more information. Follow the touchscreen UI to start your run. Note that you must have a DNA Sequencing Kit and SMRT® Cells for standard sequencing.

For Research Use Only. Not for use in diagnostic procedures. © Copyright 2015 - 2017, Pacific Biosciences of California, Inc. All rights reserved. Information in this document is sub-ject to change without notice. Pacific Biosciences assumes no responsibility for any errors or omissions in this document. Certain notices, terms, conditions and/o r use restric-tions may pertain to your use of Pacific Biosciences products and/or third p arty products. Please refer to the applicable Pacific Biosciences Terms and Conditions of S ale and to the applicable license terms at http://www.pacificbiosciences.com/licenses.html. Pacific Biosciences, the Pacific Biosciences logo, PacBio, S MRT, SMRTbell and Iso-Seq are trademarks of Pacific Biosciences. BluePippin and SageELF are trademarks of Sage Science, Inc. NGS-go and NGSengine are trademarks of GenDx. All other trademarks are the sole property of their respective owners.
