# Supplementary material for: High-throughput amplicon sequencing of the full-length 16S rRNA gene with single-nucleotide resolution
Source: Nucleic Acids Res. 2019 Jul 3;47(18):e103. doi: 10.1093/nar/gkz569 (PMC6765137; doi:10.1093/nar/gkz569)
Supplement: gkz569_Supplemental_Files [file gkz569_supplemental_files.zip › Supplementary_Figure_1.pdf]

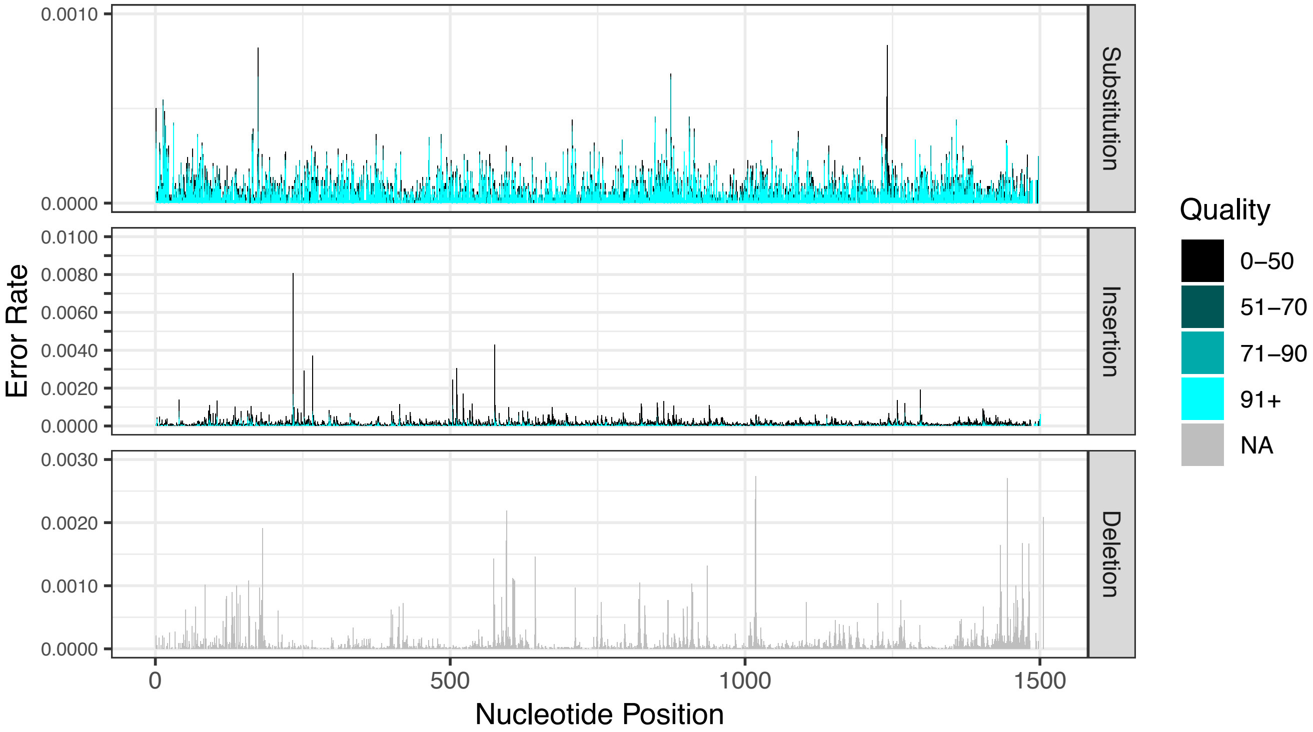

Supplementary Figure 1. Error rates in PacBio CCS amplicon reads from the Zymo mock community as a function of error type, nucleotide position and quality score. The rate of substitutions (top), insertions (middle), and deletions across all non-chimeric and non-contaminant reads from the Zymo mock community are shown. Lower quality bases are plotted in darker colors. There is no quality score associated with deletions, as such errors indicate the absence of the corresponding nucleotide in the sequencing read.
