# Supplementary material for: High-throughput amplicon sequencing of the full-length 16S rRNA gene with single-nucleotide resolution
Source: Nucleic Acids Res. 2019 Jul 3;47(18):e103. doi: 10.1093/nar/gkz569 (PMC6765137; doi:10.1093/nar/gkz569)
Supplement: gkz569_Supplemental_Files [file gkz569_supplemental_files.zip › Supplementary_Table_1.docx]

|  | **Sample** | **CCS** | **Primers** | **Filtered** | **Denoised** |
| --- | --- | --- | --- | --- | --- |
| **Mocks**  (S/P1-C1.2) | Zymo | 77453 | 73057 | 69367 | 69261 |
|  | BEI | 78328 | 73939 | 69963 | 69911 |
| **Human Fecal Samples, Replicate 1**  (S/P2-C2/5.0) | R11_1 | 16195 | 12955 | 10307 | 10212 |
|  | R3_1 | 24657 | 18588 | 15043 | 14972 |
|  | R3_2 | 22799 | 18663 | 14939 | 14772 |
|  | R3_3 | 14675 | 11838 | 9447 | 9401 |
|  | R9_1 | 25306 | 19923 | 16052 | 15935 |
|  | R9_1B | 23003 | 18362 | 14630 | 14443 |
|  | R9_2 | 19073 | 15186 | 12158 | 11906 |
|  | R9_3 | 16315 | 12641 | 10115 | 9735 |
|  | R9_4 | 12438 | 10053 | 8117 | 7816 |
| **Human Fecal Samples,**  **Replicate 2**  (S/P3-C3/5.0) | R11_1 | 19627 | 17436 | 17147 | 16974 |
|  | R3_1 | 30983 | 26605 | 26190 | 26088 |
|  | R3_2 | 27505 | 24477 | 24081 | 23856 |
|  | R3_3 | 18783 | 16738 | 16474 | 16382 |
|  | R9_1 | 32133 | 28623 | 28171 | 27951 |
|  | R9_1B | 28156 | 24832 | 24453 | 24228 |
|  | R9_2 | 24760 | 21759 | 21411 | 21071 |
|  | R9_3 | 21621 | 18701 | 18429 | 17945 |
|  | R9_4 | 15169 | 13481 | 13258 | 12864 |

**Supplementary Table 1. Reads retained at each step of our computational processing pipeline.** Three categories of samples were included in this manuscript: mock community samples, and the first and second replicate samples derived from the human fecal specimens. The mock community samples were sequenced on dedicated Sequel cells, while the human fecal samples were 12-fold multiplexed. The sequencing chemistry used for each set of samples is indicated in parentheses. CCS: PacBio CCS reads that met the default *minPasses=3* and *minPredictedAccuracy=0.999* thresholds for inclusion. Primers: PacBio CCS reads in which the forward and reverse primer sequences were detected with at most two mismatches each. Filtered: PacBio CCS with primers that passed the *maxEE=2*, *minLen=1000* and *maxLen=1600* filtering thresholds. Denoised: PacBio CCS reads that were successfully denoised.
